# The gender gap in science: How long until women are equally represented?

**DOI:** 10.1371/journal.pbio.2004956

**Published:** 2018-04-19

**Authors:** Luke Holman, Devi Stuart-Fox, Cindy E. Hauser

**Affiliations:** School of BioSciences, University of Melbourne, Parkville, Victoria, Australia; Indiana University Bloomington, United States of America

## Abstract

Women comprise a minority of the Science, Technology, Engineering, Mathematics, and Medicine (STEMM) workforce. Quantifying the gender gap may identify fields that will not reach parity without intervention, reveal underappreciated biases, and inform benchmarks for gender balance among conference speakers, editors, and hiring committees. Using the PubMed and arXiv databases, we estimated the gender of 36 million authors from >100 countries publishing in >6000 journals, covering most STEMM disciplines over the last 15 years, and made a web app allowing easy access to the data (https://lukeholman.github.io/genderGap/). Despite recent progress, the gender gap appears likely to persist for generations, particularly in surgery, computer science, physics, and maths. The gap is especially large in authorship positions associated with seniority, and prestigious journals have fewer women authors. Additionally, we estimate that men are invited by journals to submit papers at approximately double the rate of women. Wealthy countries, notably Japan, Germany, and Switzerland, had fewer women authors than poorer ones. We conclude that the STEMM gender gap will not close without further reforms in education, mentoring, and academic publishing.

## Author summary

In most fields of science, medicine, and technology research, men comprise more than half of the workforce, particularly at senior levels. Most previous work has concluded that the gender gap is smaller today than it was in the past, giving the impression that there will soon be equal numbers of men and women researchers and that current initiatives to recruit and retain more women are working adequately. Here, we used computational methods to determine the numbers of men and women authors listed on >10 million academic papers published since 2002, allowing us to precisely estimate the gender gap among researchers, as well as its rate of change, for most disciplines of science and medicine. We conclude that many research specialties (e.g., surgery, computer science, physics, and maths) will not reach gender parity this century, given present-day rates of increase in the number of women authors. Additionally, the gender gap varies greatly across countries, with Japan, Germany, and Switzerland having strikingly few women authors. Women were less often commissioned to write ‘invited’ papers, consistent with gender bias by journal editors, and were less often found in authorship positions usually associated with seniority (i.e., the last-listed or sole author). Our results support a need for further reforms to close the gender gap.

## Introduction

Although women are increasingly studying Science, Technology, Engineering, Mathematics, and Medicine (STEMM) subjects at university, women comprise a minority of senior staff, are less often trained in elite research groups, are promoted more slowly, and are more likely to leave STEMM careers [1–3]. Academic publications are the primary means of disseminating scientific knowledge and the principal measure of research productivity [4] and thus influence the career prospects and visibility of women in STEMM. Author lists of these publications also provide information on the gender ratio of people working in a given field. For these reasons, at least 61 studies have estimated the gender ratio of authors on academic publications (S1 Table). Of these, 52 used manual data collection (e.g., reading author lists), limiting their scope, while 9 used computational approaches, producing enough data to address more complex questions. For example, computational studies have so far mapped differences in gender ratio across research disciplines [5,6], compared geographic regions [6,7], revealed biases in citation rate [7–9], and shown that women tend to do a greater share of experimental work [10]. Although there is a consensus that the STEMM gender gap is shrinking (S1 Table), to our knowledge, no study has used formal modelling to predict when the gap will close, so it remains unclear when parity will be reached given present rates of change. Performing this analysis is necessary to identify disciplines that will retain an imbalanced gender ratio without additional interventions and may help to uncover previously unrecognised biases. We aimed to determine the gender and country of affiliation for each author on every publication listed in the PubMed database and the arXiv preprint server (see Methods). Together, PubMed and arXiv index around 30 million articles from the medical and life sciences, chemistry, physics, mathematics, computer science, and certain branches of engineering. We managed to assign gender with ≥95% confidence to 35.5 million authors from 9.15 million articles indexed on PubMed (2002–present) and to 1.1 million authors from 0.5 million arXiv preprints (1991–present). We obtained sufficient data to measure the author gender ratio, its rate of change, and the expected number of years to reach gender parity for 4,720 journals and 119 arXiv sub-categories (S1, S2 and S3 Data). Our web app (https://lukeholman.github.io/genderGap/) allows one to explore the PubMed data and view the past, present, and projected future gender ratio for approximately 25,000 combinations of research discipline, journal, authorship position, and country. We also confirmed that the number of women authoring research papers is a reliable predictor of the number of women working in each discipline and that the gender assigned by our computational methods to author names was correct roughly 99.7% of the time (see Methods).

## Results and discussion

### The changing nature of the gender gap

**Fig 1:**
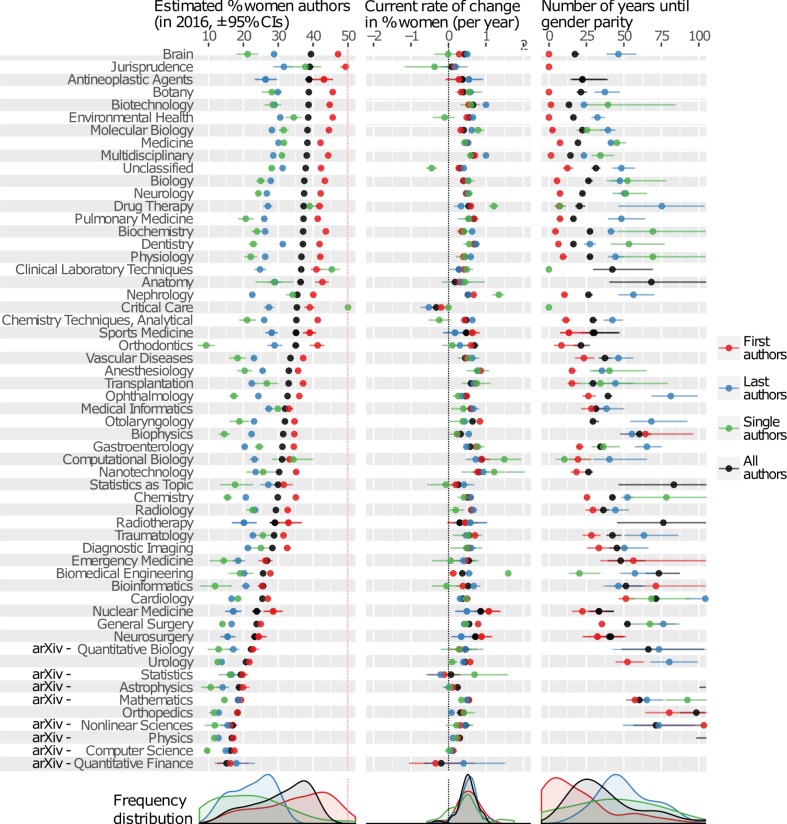
The panels show the current author gender ratio, its rate of change per year, and the estimated number of years until the gender ratio comes within 5% of parity (all parameters estimated by fitting Eq 1 to the data using maximum likelihood). The colours correspond to different authorship positions, and the error bars show 95% confidence intervals estimated by bootstrapping. For clarity, the x-axis of the third panel is truncated at 100 years. Missing data in the third panel indicate either: A) the field is never projected to reach parity, B) parity is projected to be reached in >100 years, or C) the data do not allow us to ascertain whether the percentage of women authors is presently rising or falling (full details in S1 Data). The eight disciplines using data from arXiv are marked, and the remaining disciplines are from PubMed. The data underlying this figure can be found in S1 Data.

**Fig 2:**
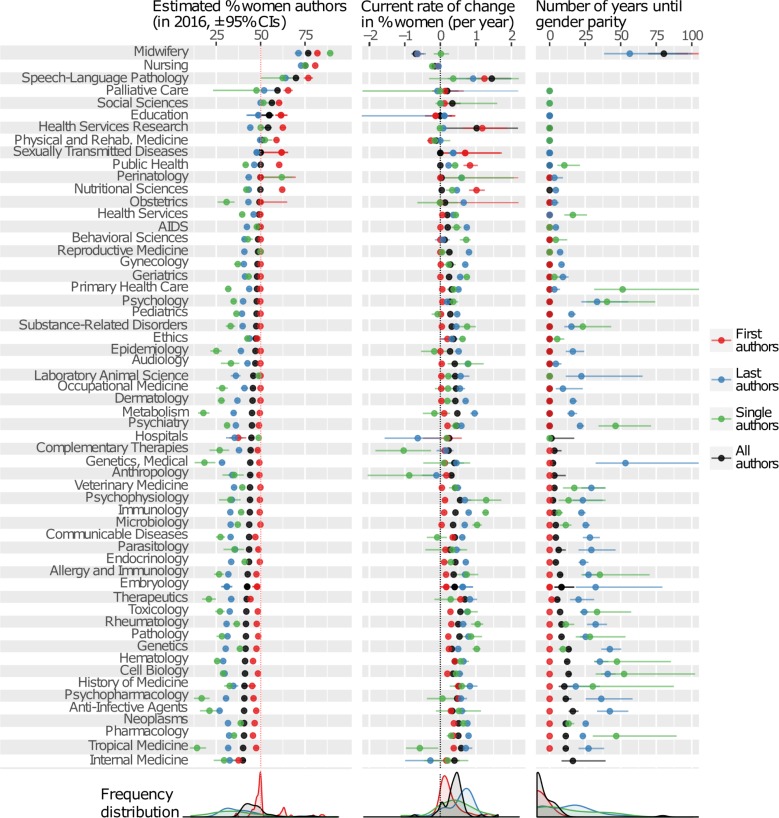
The same information as in Fig 1, for the remaining research disciplines. The data underlying this figure can be found in S1 Data.

Figs 1 and 2 reveal that 87 of the 115 disciplines examined have significantly fewer than 45% women authors, 5 have significantly more than 55%, and the remaining 23 are within 5% of gender parity. Topics such as physics, computer science, mathematics, surgery, and chemistry had the fewest women authors, while health-related disciplines like nursing, midwifery, and palliative care had the most. Of the gender-biased disciplines, almost all are moving towards parity, though some are predicted to take decades or even centuries to reach it. Nursing, midwifery, and critical care were the only disciplines in which men authors are becoming significantly more common. The PubMed categories Social Sciences (which contains predominantly Social Work journals) and Speech-Language Pathology currently have >50% women authors and are becoming significantly more female-biased. Comparable information for dozens of arXiv subcategories is shown in S1–S7 Figs: gender ratio varies by up to 20% between subfields of physics, mathematics, and computer science.

In almost all disciplines examined, women were substantially underrepresented as the last-named author in the author list and as single authors and overrepresented as first authors (contradicting prior studies [5–7]) relative to the overall author gender ratio. A small minority of journals bucked the overall trend and had fewer women first authors than expected, rather than more; these journals were predominantly well-known, prestigious titles such as *Nature*, *Lancet*, *New England Journal of Medicine*, and *BMJ* (S8 Fig). In most disciplines represented in our dataset, prevailing conventions regarding authorship order mean that first authors are usually early career researchers, while last authors tend to be comparatively senior [11]. Thus, these results suggest that early career researchers are more likely to be women and senior researchers more likely to be men, relative to the overall gender ratio of the discipline in question, consistent with United States-specific survey data showing that the underrepresentation of women is highest among senior academics [2]. In some fields, the convention is for the authors to be listed alphabetically by surname. Publications in our dataset using the alphabetical surname convention would tend to dilute the overall difference in gender ratio between first and last authors, meaning that the true difference in gender ratio between early career and senior researchers might be greater than our results suggest.

The underrepresentation of women as last authors and single authors probably has multiple, complex causes. Firstly, the number of women graduates was lower in the past, when today’s senior researchers were training (termed ‘demographic inertia’ [2]). However, Shaw and Stanton [2] used demographic data to show that demographic inertia can only partly explain the present shortage of senior women researchers in the US. Shaw and Stanton’s results (among others, e.g., [12]) point to a second reason for the dearth of senior women in STEMM: that women are more likely than men to leave STEMM careers before progressing to senior positions. A common metaphor for this issue is the ‘leaky pipeline’, which likens a STEMM career to a series of connected pipes (e.g., PhD student, junior researcher, group leader) that ‘leaks’ greater numbers of women than men at particular junctures [2]. A third possibility is that if women progress to research leadership roles more slowly than men—for example, due to facing extra challenges inside and outside the workplace [3,13,14]—the average woman would have a higher ratio of first to last author publications over her career than the average man [15]. A fourth possibility is that discussions over authorship are influenced by gender, such that women are less likely to be offered, or to request, the last author position [16]. Finally, students and other junior researchers might be less likely to select women supervisors, e.g., because equivalent achievements by women are judged less favourably [17,18] or because senior women are less often publicly celebrated as leaders in their fields [19]. Indeed, the National Academies of Science, Engineering, and Medicine (US) concluded that the deficit of women in STEMM is not because too few women enter the field or because women are less committed to their STEMM careers, but rather because ‘assumptions and stereotypes about gender operate in personal interactions, evaluative processes and departmental cultures that systematically impede women’s career advancement in academic medicine, science and engineering’ [1].

### Improvement is slowest in the most gender-biased disciplines

Worryingly, highly male-biased disciplines tended to show especially slow improvement in the gender ratio with time (Figs 1 and 2, S9 Fig). For example, the arXiv category Physics presently has around 13% women in the last author position, but this figure is only rising by c. 0.1% per year, such that the best-fitting nonlinear model (see Eq 1) predicts that it will be 258 years (95% CI 194–383) before the gender ratio of senior physicists comes within 5% of parity (S3 Data). Additionally, the difference in gender ratio between first and last authors was weakest in male-biased disciplines (Figs 1 and 2, S10 Fig); one possible explanation is that the gender gap among the newest recruits to these fields is only marginally smaller than the gender gap at senior levels. These results suggest that mostly male fields might attract fewer women graduates, lose women researchers to other careers at a faster rate, and/or have stronger gender biases that affect the relative publication rates of men and women. Thus, novel interventions appear necessary if we are to make progress in strongly gender-biased disciplines.

### What explains variance in the size of the gender gap across countries?

**Fig 3:**
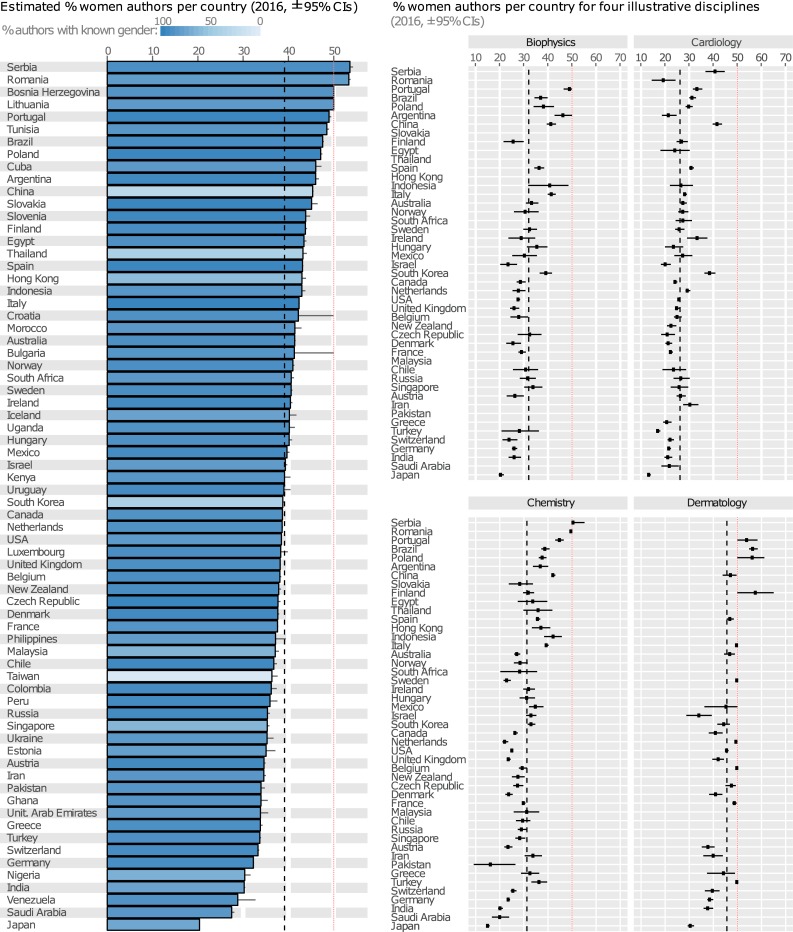
Estimated percentage of woman authors in 2016, across all authorship positions, classified by country/territory of affiliation. The dashed line shows the overall percentage of women authors across all countries, while the red line marks gender parity. Light-coloured bars indicate countries where a high proportion of authors’ genders could not be inferred from their names; gender ratios for these countries could conceivably be inaccurate (see S20 Fig). The right-hand panels show the gender ratio within four illustrative research disciplines (S10–S15 Figs show the remainder) for the 50 countries with the largest sample size, illustrating that the ordering of countries remains broadly similar within most research disciplines. The data underlying this figure can be found in S2 Data.

Across countries, the gender ratio of all PubMed-indexed authors varied by >30% (Fig 3, S11–S18 Figs). Among the major research-producing countries, the STEMM gender gap was especially pronounced in Japan, Germany, and Switzerland. The most gender-equitable countries spanned Europe, South America, and Africa. Using data on gender equality and development collected by the United Nations, we found that countries in which children of both sexes attend school longer have more women authors, while countries with higher per capita income have fewer women authors (S19 Fig; S2 and S3 Tables). Life expectancy, adolescent birth rate, percentage of women in parliament, and the education and labour force gender gaps were not significantly correlated with the STEMM gender gap. Though correlational, these results imply that wealth does not necessarily diminish gender inequality in the STEMM workforce, though access to education might. Cultural and historical factors are challenging to meaningfully capture in this type of analysis, but we suspect that they play a major role.

### Inequality persists even in fields with many women authors

Variation in author gender ratio between journals could reveal underappreciated biases against women and allow us to ask whether women’s research is published in equally visible, prestigious fora. For example, so-called ‘high impact’ journals tend to be well cited, widely read, and prestigious, and the same is true of many journals that specialise in reviews (e.g., *Nature Reviews* or Cell Press *Trends* journals). Both journal types also commonly reject more manuscripts, or publish more papers by invitation, which might disadvantage women [14,17]. By contrast, some Open-Access (OA) journals accept a comparatively high proportion of papers and are considered by some researchers to be less prestigious (debated in [20]). We therefore tested whether impact factor and journal type correlated with author gender ratio.

**Fig 4:**
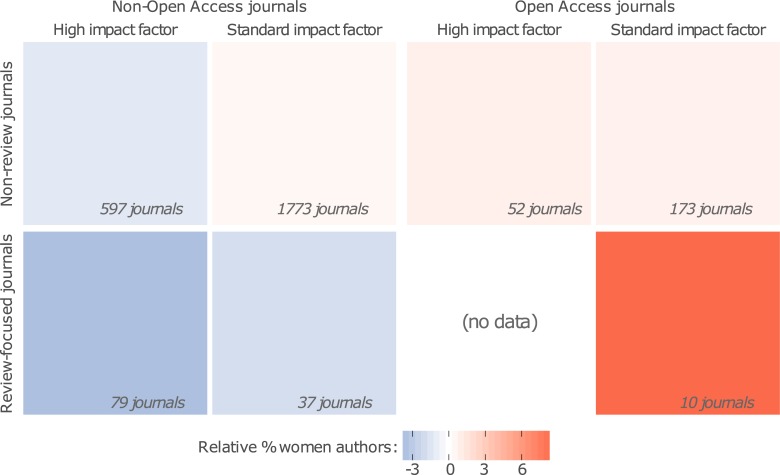
Average effects of journal impact factor, and of being a review-focused journal or OA journal, on the frequency of women authors. The colour shows the relative frequency of women authors (i.e., percentage of women authors in the journal, minus the percentage of women authors for the discipline to which that journal belongs), averaged across journals of each type. Thus, bluer (redder) squares denote journal types with an excess of men (women) authors after controlling for differences in gender ratio across disciplines. For illustrative purposes, ‘High impact’ journals are defined here as those with an impact factor in the top 25% for their discipline (although impact factor was treated as a continuous variable during statistical analysis; S5 Table). Inset numbers give the number of journals used to calculate the average relative gender ratios. The data underlying this figure can be found in S1 Data. OA, Open-Access.

Journal impact factor (standardised by discipline) negatively correlated with the proportion of women authors. Review-focused journals also had fewer women authors than non-review-focused journals, and there were more women authors in OA than non-OA journals, particularly within review journals (Fig 4; S5 Table). These results imply that women are disadvantaged and suggest remedial strategies. Prestigious journals tend to reject many submissions without peer review, and editors are usually aware of authors’ names (and thus genders), even for journals that use double-blind peer review. Gender bias has been implicated in nonexperimental studies of peer review [21] and experimentally demonstrated in other academic contexts [14,17], suggesting a need for double-blind editing and review.

We hypothesized that women may be invited to submit academic papers less often than men [22], given that this is the case for invited keynote lectures at some conferences [19]. A gender bias in the rate of invitations would directly contribute to the author gender gap and to differences in gender ratio between journals. By applying simulations to 3,067 invited papers detected in our dataset, we estimate that men are roughly 1.7–2.1 times more likely than women to be invited to submit papers (see Methods; S4 Table). The gender ratio among authors of invited papers is even more male-biased than the gender ratio of last authors or single authors (S4 Table). This suggests a need to scrutinise editorial practices [22], appoint women editors [23], and implement gender targets when using an invitation-based publishing model [19].

Another explanation for the elevated gender disparity we observed in higher-impact journals is that women submit a lower proportion of their manuscripts to prestigious journals. A recent study, upon finding that women’s papers passed peer review more frequently than men’s, hypothesised that women practice ‘better targeting of papers to a journal’ [24]. A more pessimistic hypothesis is that women are not encouraged to aim high by colleagues and mentors [1] or do not try because they believe themselves to have a lower chance of success [25]. These issues could be addressed via mentoring programs for staff and team leaders [1] and by taking steps [1,19] to promote women role models [26]. Because the variables examined here all potentially affect citation rate, our results partially explain previous reports [7–9] that women’s papers are cited less often than same-discipline papers written by men. The ‘citation gap’ would likely shrink if barriers to women publishing highly visible papers, e.g., invited reviews in prestigious journals, were removed.

### Conclusions

In closing, our data reveal an authorship gender gap across STEMM, which is likely to remain for generations in certain fields. On a positive note, many disciplines are already close to having equal numbers of men and women authors, others are making steady progress towards parity, and no male-biased disciplines displayed a clear decline in the number of women authors. Women were especially common in the first authorship position across most fields, implying that, worldwide, increasing numbers of women are starting careers in STEMM. However, some STEMM disciplines (e.g. Physics, Computer Science, Surgery) clearly require additional interventions if parity is to be reached this century, and women were strongly underrepresented in authorship contexts (namely last, single, and invited authors) that are typically occupied by senior researchers, even in fields with gender parity in the overall pool of authors.

We suggest that efforts to recruit and retain women in STEMM must be wide-ranging and could include dispelling the common but poorly evidenced belief that there are innate gender differences in STEMM aptitude [27,28]; reforming academic publishing and peer review [21]; ensuring women have equal access to informal professional networks [29]; affording greater recognition of the extra demands outside the workplace that traditionally fall on women when assessing candidates’ achievements [13]; guaranteeing women equal resources at work [9]; providing better access to parental leave [2,29] and additional provisions to help people return to work following a career break [30]; striving for a representative gender ratio of invited speakers at academic conferences [19]; and affirmative action during hiring.

Our dataset has been publicly archived, and we hope it will prove useful to researchers, policy makers, journals, and scientific societies. Follow-up studies using the dataset could search for additional predictors of author gender ratio. For example, one could measure the impact of double-blind peer review or invitation-based submission models on the proportion of women authors. One could also seek to identify cultural and sociological factors common to countries with good gender balance in STEMM or measure the impact of policies implemented by countries, journals, or granting agencies aimed at promoting women in science.

## Methods

### Overview of methods

The objective of our study was to assess the past, present, and future gender ratio of authors publishing in many different fields of STEMM. In particular, we sought to identify fields in which parity will not be reached for many years, assuming that present trends continue. The study began by downloading the author list for every single publication or pre-print indexed on the PubMed and arXiv databases. We then used the genderize.io database to assign genders to authors based on their given names and, where possible, determined the country in which each author was based from their academic affiliation. For the PubMed data, each journal was assigned to an academic discipline, using PubMed’s own classifications where possible. For each journal, discipline, and arXiv category, we fit a model to the data to estimate the present-day author gender ratio, its rate of change, and when (or if) gender parity will be reached. Additionally, we conducted several analyses searching for variables that explain variation in the author gender ratio. For example, we tested whether journal impact factor correlates with gender ratio, compared the author gender ratio of invited papers to that of papers submitted without an invitation, and searched for country-level correlates of the author gender ratio using metrics of equality and development collected by the United Nations.

To facilitate easy access to our data, we have produced an interactive web application that allows one to view the past, present, and projected future author gender ratio for different combinations of journal, research discipline, country, and authorship position (https://lukeholman.github.io/genderGap/). The complete dataset is archived as a SQLite3 database at the Open Science Framework (https://osf.io/bt9ya/)). Scripts used to collect and analyse the data, as well as a compact spreadsheet summarising our dataset, is archived at https://github.com/lukeholman/genderGapCode. The JavaScript and .json file underlying the web app is available at https://github.com/lukeholman/genderGap.

### Retrieving data from PubMed and arXiv

We downloaded a local copy of PubMed’s MEDLINE database on 20 August 2016 using a shell script. For each unique article, we used R scripts to process the XML database entry and extract the first-listed given name of every author (or the second given name, if the first was a single letter, as in B. Rosemary Grant). We also retrieved the paper’s title, the journal name, the addresses for all authors, the DOI, and the date on which the article was added to PubMed (to the nearest day). From each author's affiliation, we attempted to determine the country in which they were based by pre-processing the addresses using *libpostal* (github.com/openvenues/libpostal), followed up with custom R scripts that searched for the country name (or inferred it from the state or city). Some major research-producing regions, e.g., Taiwan and Hong Kong, were left separate from their countries. We filtered the PubMed data to exclude articles without authors and removed articles from journals that had fewer than 100 articles indexed on PubMed (inspection suggested that these were usually not standard academic journals).

On 17 October 2016, we downloaded data for every preprint on arXiv using the R package *aRxiv* (github.com/ropensci/aRxiv). For each article, we recorded ID, initial publication date, the authors’ given names, and the major and minor research specialties (which are selected by the authors). Preprints lacking authors were discarded.

### Inferring gender from authors’ given names

Gender was assigned to given names using the genderize.io web server, which uses a large database of name–gender associations assembled by trawling social media websites (>200,000 given names, with country-specific name–gender associations for approximately 80 countries). Whenever we knew an author’s country of affiliation, we specified the country when querying genderize.io to determine the gender of their name; this should reduce the misclassification rate (e.g., people named Kim are typically male in Denmark but female in the US).

For each name, genderize.io returns a number (the ‘gender score’) corresponding to the proportion of people with that name in the genderize.io dataset who are men or women; for example, 7% and 59% of people named ‘Chris’ and ‘Robin’ are women, respectively. Given the abundance of data, we elected to deal with this ambiguity by simply excluding names that were not associated with one gender with ≥95% frequency in the genderize.io database. This simple approach preserved 92.4% of the dataset (3.7 million authors were excluded, leaving 35.5 million). The gender score of the 3.7 million excluded names was not appreciably skewed towards zero or one (gender score mean: 0.47, median: 0.49), indicating that this procedure did not bias our estimated gender ratios.

To verify our computationally assigned gender dataset, we compared it to a similar, manually curated dataset from an in-prep paper by Michael McCarthy and colleagues (University of Melbourne), in which author gender was ascertained via Google searches. Of the 372 authors that appeared in both datasets and were assigned a gender by us, there was only one error (a man misclassified as a woman). Thus, we estimate our gender misclassification rate as 1/372 = 0.3% (95% CI 0%–1.7%). The manually collected data contained an additional 29 authors whose gender was left ‘unclassified’ in our dataset, either because their given names were associated with one gender <95% of the time (18 authors), were written as initials (6 authors), or were absent from genderize.io (5 authors). The manual dataset estimated the author gender ratio as 23.4% women (*n* = 401, 95% CI 19%–28%), while we estimated it as 24.4% (*n* = 372, CI 20%–29%); thus, the accuracy and precision of our method was essentially the same as that afforded by manual assignment. See also S21 Fig for additional evidence that our methods produced accurate estimates of the gender ratio. Lastly, we note that within any given dataset, male-to-female and female-to-male misclassification errors will partially cancel out (e.g., 10 misclassified men and 6 misclassified women would have the same impact on the author counts as 4 misclassified men, making 16 errors behave like 4), reducing the impact of errors.

### Metadata regarding each journal and country

We next classified PubMed-indexed journals into STEMM research disciplines. Because subjective assignment of journals to disciplines could theoretically introduce bias, we used PubMed’s preexisting classifications wherever available. PubMed maintains a database of information on many (but not all) of the journals that they index (ftp://ftp.nlm.nih.gov/online/journals/), and in some cases, PubMed curators have manually assigned journals to a research discipline. Additionally, PubMed have assigned Medical Subject Heading (MeSH) terms to many journals; MeSH terms derive from text mining and describe a journal's content (nlm.nih.gov/mesh). We assigned a single discipline to each journal using the manually assigned heading (if present), a random PubMed category listed in the MeSH terms (if the former was not present), and, as a last resort, keywords in the journal's title (e.g., journals with ‘Kidney’ in the title were added to the PubMed category ‘Nephrology’). Assignment was done blind to the gender data; we focused on categorising journals that contained a lot of data and stopped once further categorisation became laborious, leaving 579 journals (median: 293 papers each) as ‘Unassigned’.

Although using the PubMed categorisations has the advantage of being objective (preventing us from inadvertently biasing the results), a limitation of this approach is that the various subfields of medicine are precisely categorised, but other disciplines are not. For example, PubMed indexes many ecology journals but classifies them under broader headings, such as Biology. Readers interested in a discipline that is missing from our set can use our web app to search for data from relevant journals.

We also used data from the Directory of Open Access Journals (www.doaj.org) to classify journals as OA or non-OA. Only journals in which all content is freely accessible were classified as OA; journals that allow OA publishing for an additional fee were classified as non-OA. We also obtained the 3-year impact factor of each journal tracked by Clarivate Analytics (formerly the Institute for Scientific information [ISI]). Although we recognise that a journal’s impact factor is a weak predictor of the quality or citation count of any given paper in that journal, impact factor is (for better or worse) a widely used metric of journal prestige and the productivity of individual researchers, and journals with the highest impact factor in their discipline tend to be more well-established and widely read [31]. Thus, it is worthwhile to determine whether the author gender ratio differs between high and low impact factor journals, since this could affect the relative visibility of women authors or their perceived research productivity. Journals with a title containing the words “Review(s)” or “Trends” were classified as review-focused journals, while the remainder were classified as non-review-focused. In initial trials, we also attempted to classify individual articles as OA or as reviews (not just whole journals), but this proved difficult to do reliably. Our journal-level analyses are probably conservative, because OA or review-type articles that are published in predominantly non-OA and non-review-focused journals will be misclassified (and vice versa), reducing the observed difference in author gender ratio.

We obtained country-level metrics on gender inequality, wealth, and development from the United Nations Human Development Report 2015 (http://hdr.undp.org/en/global-reports). We downloaded all the available UN variables, except the metrics of poverty (which are unavailable for most major research-producing countries) and the human development index and maternal mortality rate (because these were >90% correlated with other variables in the set), resulting in seven predictors. We focused on metrics of gender equality and development due to our a priori expectation that these might be key predictors of the number of women in science.

### Estimating and predicting the gender ratio

We analysed four types of authorship. We recorded the number of men and women listed as A) first authors of multiauthor articles, B) last authors of multiauthor articles, C) authors of single-author articles, D) authors covering all authorship positions of both single- and multiauthor articles (termed ‘overall’).

To estimate the present-day gender ratio and its rate of change, we assumed that the proportion of women authors (*p*) scales with publication date via the logistic function:

$$p=\frac{e^{0.5rt}}{2\;e^{0.5rt}+c}$$ (1)

where *t* is the date (to the nearest day, expressed as a decimal number of years before or after 1 January 2000), *r* controls the steepness of the curve, and *c* varies its inflection point. This model assumes that the relationship between gender ratio and time is sigmoidal and progresses monotonically either towards gender parity or the complete disappearance of one gender. The model thus allows for nonlinear rates of change in the gender ratio (which were common in our data) and accommodates our strong prior expectation that gender-biased disciplines that are heading towards parity are likely to eventually plateau at parity (as opposed to overshooting parity or reversing direction, as occurs if one uses alternative methods such as quadratic linear regression or generalized additive models). Some combinations of *r* and *c* also allow the relationship between *t* and *p* to appear almost flat over long timescales, allowing the model to accommodate an essentially unchanging gender ratio.

We found the maximum likelihood pair of *r* and *t* values for a given set of data using R’s *optim* function and substituted them into the above formula to estimate the gender ratio on the present day (i.e., 20 August 2016, the date when the data were retrieved from PubMed). We also estimated the present-day rate of change in the gender ratio (by differentiating Eq 1 with respect to *t*) and the projected number of years until gender parity. We estimated the 95% confidence intervals on these 3 variables using bootstrap resampling of the data (*n* = 1,000).

To ensure accurate prediction, we only predicted the gender ratio, its rate of change, and time-to-parity on subsets of the data that contained at least 100 papers and which contained at least 50 authors of known gender per year for five or more years.

### The PubMed and arXiv datasets

Our PubMed gender data are almost entirely from 2002–present, because earlier PubMed entries generally list authors’ initials instead of their given names. arXiv was founded in 1991, though 97% of entries were from 1998 or later. Programmatic gender assignment was mostly successful: 48.7 million authors were listed in the subset of PubMed articles that listed at least one author’s given name, and we were able to assign gender with ≥95% confidence to 35.5 million of these (i.e., 73%, or 76% if discounting authors who only gave their initials; S20 Fig). Because <3% of the authors in our PubMed dataset used initials rather than their full name, a gender difference in the tendency to publish under one’s initials would not appreciably skew our results.

We detected 116 countries and territories in authors’ affiliations. Gender was assigned with ≥95% confidence to at least 70% of authors for 96/116 countries (S20 Fig), though gender assignment was frequently unsuccessful for authors with affiliations in some East Asian countries, due to a high frequency of (Romanized) given names that are commonly used by both men and women. However, for most countries, failures to assign gender were infrequent enough that they could not add significant bias, even in the unlikely event that programmatic gender assignment was substantially more fallible for names of one particular gender.

Gender was inferred with ≥95% confidence for 1.18 million authors (61.1%) from 538,688 arXiv preprints. This lower success rate reflects the fact that arXiv does not mandate a consistent style for presenting authors’ names, and more authors only provided their initials—the success rate was 85.0% when excluding these authors.

### Identifying correlates of author gender ratio across countries

The following analysis was conducted on the subset of authors whose genders and countries were known, and the authors were from a country listed in the UN dataset (*n* = 22,510,436 authors). We fit a linear mixed model with the percentage of women authors as the response variable, because initial tests showed that binomial generalized linear mixed models (with author gender as the response variable) did not converge (using the *lmer* and *glmer* functions in the lme4 package for R, respectively). The model had the following formula:

$$\%\;women\;authors∼a_{1}Position+a_{2}\mathrm{Date}+a_{3}x_{1}+a_{4}x_{2}+a_{5}x_{3}+a_{6}x_{4}+a_{7}x_{5}+a_{8}x_{6}+a_{9}x_{7}+\left(Date|Journal\right)+\left(Date|Discipline\right)+\left(Date|Country\right)$$

where *x*_1_–*x*_7_ are the seven UN predictor variables and terms in parentheses are random effects. ‘Date’ is publication date to the nearest year (treated as a continuous variable), and ‘Position’ is authorship position (first, last, middle, or single; first authors were treated as the reference level, and ‘middle’ authors were those whose names appeared between the first and last authors of multiauthor papers). To standardise the units of the model coefficients, publication date and all the UN predictor variables were transformed to have mean 0 and variance 1. The notation ‘Date|Journal’ indicates that ‘Journal’ was treated as a random intercept and publication date as a random slope. Thus, the model estimates the fixed effects (*a*_1_–*a*_9_) after controlling for differences in the gender ratio between journals, disciplines, and countries, where these differences potentially vary linearly with time. The marginal *R*^2^ of the model (i.e., the proportion of variance explained by the fixed factors) was 0.08, and the conditional *R*^2^ (the proportion of variance explained by both fixed and random factors) was 0.66.

### Testing whether invited papers have an atypical author gender ratio

We searched for papers that were published by invitation by examining the titles of all papers in the PubMed dataset. We defined a paper as ‘invited’ if its title began with the word ‘invited’ followed by another word, such as article, comment(ary), discussion, editorial, essay, review, or paper; these second words were selected based on manual inspection of every title beginning with ‘invited’. Note that this analysis presumably misses very many invited papers that do not have the word ‘invited’ in their titles. This limitation means that our analysis probably underestimates the true difference in gender ratio between invited and noninvited papers, but it seems unlikely that it could produce a spurious difference in gender ratio.

We then wrote a simulation to test whether these invited papers had significantly fewer women authors than would be expected under the null hypothesis that invited papers have the same gender ratio as for all papers. In short, the simulation uses information from our dataset to ask, ‘Is the frequency of women authors on invited papers lower than expected, after controlling for variation in gender ratio due to journal, publication date, and (optionally) authorship position?’ Modelling these sources of variation is necessary because the frequency of invited papers differs across journals and across time, and our sample of invited papers contains an atypically high frequency of single-author papers, all of which could produce a spurious correlation between invitation and gender if not controlled for.

To obtain the null expected author gender ratio for the 3,067 invited papers, we generated 10,000 random datasets, each of which had the same number of papers and same distribution of authors per paper as our sample of invited papers but which had randomly generated author genders. Each author’s gender was randomly generated by sampling from a binomial distribution with probability *p*(Woman | Journal, Date, Position), i.e., the probability of the focal author being a woman, given the journal in which that person was publishing, the date on which the paper appeared, and the authorship position of the focal person (i.e., first, last, middle, or single author—we assumed that the gender ratio for middle authors was the same as the overall gender ratio, unless otherwise stated). We obtained this probability by substituting the values of *r* and *c* estimated using the data (i.e., invited and noninvited papers together) into Eq 1. This gave us the expected distribution of gender ratios for the invited papers, under the null hypothesis that men and women are invited equally often, given the simulation’s assumptions. Because the results are sensitive to the simulation’s assumptions, we used four sets of assumptions of varying strictness when defining *p*(Woman), which are outlined in S4 Table. The four assumptions all gave a qualitatively identical answer, namely that authors of invited papers are more likely to be men relative to our predictions under the null models.

### Can author gender ratio be used to infer the gender ratio of researchers?

Differences in author gender ratio between disciplines, journals, and countries could be due to variation in the number of women researchers and/or to variation in the relative publication rates of men and women. It is also conceivable that we made errors when measuring the author gender ratio that escaped detection. To address these concerns, we tested whether the author gender ratio that we estimated from PubMed was correlated with estimates of the number of women working in various subdisciplines derived from surveys conducted by the US’s National Science Foundation (NSF). Assuming that research disciplines in which many women work have more women authors and that our methods lack errors, we predict a strong positive correlation between the NSF data and our own.

The NSF’s *Survey of Graduate Students and Postdoctorates in Science and Engineering* provides data on the gender ratio of PhD students and postdoctoral researchers working in a number of disciplines (Tables 21, 22, and 38 at https://ncsesdata.nsf.gov/gradpostdoc/). Unfortunately, we could not use NSF’s data on more senior researchers, because the NSF chose not to group senior researchers into the same fine-scale disciplines as the ones they use for early career researchers (senior scientists were instead grouped into much broader categories, e.g., ‘Life Sciences’). Blind to the gender data, we manually matched each NSF research category with a similar discipline used by PubMed and tested for a correlation between the author data (using US-based authors only) and the NSF gender data for PhDs and postdocs using linear regression.

We found that our gender ratio data were tightly correlated with the proportion of women PhD students and postdocs as measured by the NSF (*R*^*2*^ = 0.48–0.64; S21 Fig); in particular, the gender ratio among first authors was within 5% of the gender ratio of postdocs in most disciplines. Thus, it is indeed possible to use authorship data to reliably compare the gender ratios of different STEMM disciplines, at least for early career researchers, and we found no evidence that our methods introduced significant errors.

However, this analysis revealed that our PubMed data sometimes underestimated the number of women early career researchers, particularly in female-biased disciplines or when using authorship positions other than the first (S21 Fig). This implies that, among US academics, the average man has more publications than the average women (especially last- and single-author publications). Note that the average male researcher has been publishing for longer than the average female researcher [2], and S21 Fig does not account for gender differences in career length; thus, the gender difference in publication number is probably smaller than implied by the apparent shortfall in S21 Fig.

## Supporting information

S1 Fig Comparable data to Fig 1 for the subcategories within the ArXiv category *Physics*. (EPS)

S2 Fig Comparable data to Fig 1 for the subcategories within the ArXiv category *Mathematics*. (EPS)

S3 Fig Comparable data to Fig 1 for the subcategories within the ArXiv category *Computer Science*. (EPS)

S4 Fig Comparable data to Fig 1 for the subcategories within the ArXiv category *Quantitative Biology*. (EPS)

S5 Fig Comparable data to Fig 1 for the subcategories within the ArXiv category *Astrophysics*. (EPS)

S6 Fig Comparable data to Fig 1 for the subcategories within the ArXiv category *Statistics*. (EPS)

S7 Fig Comparable data to Fig 1 for the subcategories within the ArXiv category *Nonlinear Sciences*. (EPS)

S8 Fig Although women were greatly overrepresented in the first author position in the majority of journals, women first authors were underrepresented in certain journals, many of which had high impact factors. The x-axis shows journal impact factor, which has been standardised based on the discipline to which the journal belongs (using model predictions as in S5 Table). The y-axis shows the difference in the present-day gender ratio between first authors and all authors, such that positive numbers indicate that women are overrepresented as first authors.
(EPS)

S9 Fig Each point corresponds to one of the research disciplines from Figs 1 and 2. The plot shows the author gender ratio in 2016 and the change in gender ratio per year (i.e., the first two columns of Figs 1 and 2) for disciplines with <50% women. In all authorship positions, the proportion of women authors is increasing more slowly for the most highly male-biased disciplines than for those that are less male-biased. The curves and their 95% CIs are derived from generalised additive models with *k* = 3, and data points with greater sample sizes were given extra weight when calculating the curves.
(EPS)

S10 Fig Each point corresponds to one of the research disciplines from Figs 1 and 2. The x-axis shows the overall percentage of women authors in 2016 (i.e. black points in the first column from Figs 1 and 2), and the y-axis shows difference between this value and the percentage of women authors in 2016 in the first or last authorship position (i.e., the red or blue points in Figs 1 and 2). The excess of women first authors is smallest for highly male-biased disciplines, while the deficit of women last authors is almost smallest, suggesting that the gender ratio of junior and senior scientists is comparatively similar in highly biased disciplines, as one would predict if women are entering the most male-biased disciplines at lower rates. The curves and their 95% CIs are derived from generalised additive models with *k* = 3, and data points with greater sample sizes were given extra weight when calculating the curves.
(EPS)

S11 Fig The percentage of women authors, across all authorship positions, split by country and PubMed research discipline. A data point is plotted for all cases for which we recovered author gender data from at least 100 papers, and 50+ authors from at least five different years. The dashed line shows the gender ratio across all countries.
(EPS)

S12 Fig The same information as S11 Fig, for other PubMed research disciplines. (EPS)

S13 Fig The same information as S11 Fig, for other PubMed research disciplines. (EPS)

S14 Fig The same information as S11 Fig, for other PubMed research disciplines. (EPS)

S15 Fig The same information as S11 Fig, for other PubMed research disciplines. (EPS)

S16 Fig The same information as S11 Fig, for other PubMed research disciplines. (EPS)

S17 Fig The same information as S11 Fig, for other PubMed research disciplines. (EPS)

S18 Fig The same information as S11 Fig, for other PubMed research disciplines. (EPS)

S19 Fig Parameter estimates (and their 95% CIs) for publication date, authorship position, and various metrics of gender equity and development taken from the 2015 UN Human Development Reports. The parameters were scaled to mean 0 and variance 1 prior to analysis, meaning that the x-axis shows the estimated effect of increasing the predictor variable by one standard deviation. For example, countries one standard deviation above the average per capita gross national income have 5.5% fewer women authors on PubMed, after controlling for the effects of the other parameters. The factor levels of ‘Authorship position’ are expressed relative to first authors; thus, women first authors are significantly more common than are women middle, last or single authors.
(EPS)

S20 Fig We could assign gender to >70% of authors with ≥95% certainty for 96 countries, though gender assignment failed for the majority of authors in a few countries (e.g., China). The colour of the bars shows the number of authors affiliated with each country/territory in the dataset analysed in this paper on a log_10_ scale.
(EPS)

S21 Fig The percentage of women PhD students (left) and postdocs (right) recorded in surveys by the NSF is tightly correlated with the percentage of US-based women authors across research disciplines (in all four authorship positions; top to bottom panels). Each point is an NSF research category for which we had data on author gender ratio (*n* = 43), and the text gives the linear regression slope (*m*), its 95% confidence intervals, and the R^2^ value. The dashed line shows y = x. NSF, National Science Foundation.
(EPS)

S1 Table A nonexhaustive literature review of 61 previous studies that compared the number of male and female authors on scholarly publications. The data were obtained from Google Scholar searches and by inspecting the reference list of each recovered paper. Note that there are usually fewer women authors than men and that some fields are progressing towards gender parity. The ‘% female authors’ column shows the data from the last available year (if presented; otherwise, it shows the gender ratio of the overall sample).
(PDF)

S2 Table Analysis of deviance table from the mixed model of percentage of women authors that is described in the Methods, showing the results of Type III Wald chi-squared tests. Predictors that significantly correlated with percentage of women authors are shown in bold. ‘Authorship position’ is a 4-level factor with the levels first, last, middle, or single author. The remaining predictors are continuous variables, though publication date was rounded off to the nearest year.
(PDF)

S3 Table The random effect terms from the mixed model of percentage of women authors that is described in the Methods. Journal and discipline each explained about a quarter of the variation, while country explained 11%. The random slopes and intercepts were weakly negatively correlated for journal and discipline, reflecting the fact that percentage of women authors is increasing in most fields with a male-biased gender ratio and is decreasing (or increasingly more slowly) in most fields with a female-biased gender ratio.
(PDF)

S4 Table Results of four simulations all testing for a difference between the observed and expected gender ratio for the authors of invited papers, listed in the order that we regard as least to most conservative. The ‘Use last for single’ simulation generated the expected author GR of invited authors under the null hypothesis (i.e., that there is no difference in GR between invited and noninvited authors) using our estimates of how journal, authorship position, and publication date affect GR. For cases where we had no estimate of the GR of single authors, we assumed it was the same as the GR of last authors, increasing the sample size of the test. The ‘Complete records only’ method is the same, but instead of filling in missing data, it simply excludes cases for which we did not have an estimate of the GR for all authorship positions. The ‘Use last for all’ method assumes that the GR of invited authors is expected to be the same as the GR for last authors (even among first and middle authors on invited papers)—this captures our expectation that invited authors will tend to be drawn from the pool of senior researchers (which is male-biased), rather than the total population of researchers. The final method is the same, except that it assumes that the GR should be equivalent to that estimated for single authors. In all simulations, the null expected gender ratio for all invited papers was randomly generated 10,000 times, then compared to the observed gender ratio as both an absolute difference and a fold difference (*p* < 0.00001 for all four models). The 95% quantiles of this differences were used to estimate the 95% confidence limits on the difference. The final columns give the sample sizes for each method in terms of authors, papers, and journals. GR, gender ratio.
(PDF)

S5 Table Type III ANOVA table of the top-ranked model of the dataset shown in Fig 4. We identified the top model by comparing the AICc scores of the full model (containing the 3 predictors and all 2- and 3-way interaction terms) and all possible simpler models. ‘Relative impact factor’ is a continuous predictor, defined as the residuals from a model with Log_10_ IF as the response variable and research discipline as a random effect (i.e., it gives the IF after adjusting for the differences in impact factor that exist between disciplines). ‘Review journal’ and ‘OA journal’ are both 2-level factors describing whether the focal variable is a review journal or an Open Access journal. AICc, corrected Akaike Information Criterion; IF, impact factor.
(PDF)

S1 Data Gender ratio information for each journal and research discipline for which we recovered sufficient data in each of four different authorship positions (first, last, single, and overall). Each row gives the sample size in terms of authors and individual papers, followed by the estimated gender ratio at the present day (i.e., August 2016) and the associated 95% confidence limits. Next, the estimated current rate of change (in percentage of women authors per year) is given, followed by the estimated years until the gender ratio enters the range 45%–55% women authors. The columns r and c give the maximum likelihood parameter values for these parameters for the logistic curve fit to the data (Eq 1). The final column gives a qualitative description of the gender gap for each item, e.g. ‘Male-biased but improving’, or ‘Essentially at parity’.
(CSV)

S2 Data The same dataset as in S1 Data, except that each journal and discipline has been further subdivided by author country of affiliation. As before, only combinations for which we recovered sufficient data appear.
(CSV)

S3 Data Analogous information to that contained in S1 Data, for the ArXiv categories and subcategories. As before, only combinations for which we recovered sufficient data appear.
(CSV)

## Abbreviations

ISI: Institute for Scientific Information
MeSH: Medical Subject Heading
NSF: National Science Foundation
OA: Open-Access
STEMM: Science, Technology, Engineering, Mathematics, and Medicine
