# Supplementary material for: The gender gap in science: How long until women are equally represented?
Source: PLoS Biol. 2018 Apr 19;16(4):e2004956. doi: 10.1371/journal.pbio.2004956 (PMC5908072; doi:10.1371/journal.pbio.2004956)
Supplement: S1 Table — The data were obtained from Google Scholar searches and by inspecting the reference list of each recovered paper. Note that there are usually fewer women authors than men and that some fields are progressing towards gender parity. The ‘% female authors’ column shows the data from the last available year (if presented; otherwise, it shows the gender ratio of the overall sample). (PDF) [file pbio.2004956.s022.pdf]

Reference	Method	Discipline	Focus	Years sampled	Sample size	% female authors	Change over time
Caplar et al. (2016) *arXiv* 1610.08984.	Database analysis	Astronomical sciences	Astrophysics Data System (5 journals) and arXiv	1950-2015	149,741 articles	*c.* 25%	Increase
Poling et al. (1983) *The Behavior Analyst* **6**, 145-152.	Manual collection	Behavior analysis	2 journals	1958-1981	Not stated	*c.* 8-22%	Increase
Myers (1993) *The Behavior Analyst* **16**, 75-86.	Manual collection	Behavior analysis	3 journals	1978-1992	Not stated	*c.* 30%	Increase
McSweeney & Swindell (1998) *The Behavior Analyst* **21**, 193-202.	Manual collection	Behavior analysis	4 journals	1978-1997	Not stated	30-50%	Increase
McSweeney et al. (2000) *The Behavior Analyst* **23**, 267-277.	Manual collection	Behavior analysis	4 journals	1978-1997	Not stated	20-40%	Increase
Walters et al. (1990) *Research in Higher Education*, **31**, 365-7.	Manual collection	Business	27 journals	1962-1984	31,617 authors	16%	Increase
Singh & Jatoi (2008) *Journal of Cancer Education*, **23**, 192-4.	Manual collection	Cancer palliative care	5 journals	1990, 1995, 2000, 2005	460 articles, 452 first and 342 last authors	39-50%	Static
Kramer et al. (2007) *Review of Communication*, **7**, 229-40.	Manual collection	Communication	4 journals	1934-2005	>400 articles	*c.* 35%	Increasing
Bonham & Stefan (2017) *PLoS Computational Biology* 13: e1005134.	Database analysis	Computational Biology	PubMed and arXiv	2007-2016	4-500,000 articles	*c.* 15-20%	Increase
Eigenberg & Baro (1992) *Journal of Criminal Justice Education*, **3**, 293–314.	Manual collection	Criminology	5 journals	1976, 1979, 1982, 1985 & 1988	835 articles, 1,412 authors	16%	Increasing
Eigenberg et al. (1993) *Women and Criminal Justice*, **4**, 165-70.	Manual collection	Criminology	5 journals	1976, 1979, 1982, 1985 & 1988	Identical dataset to 1992 paper	16%	Increasing
Crow & Smykla (2014) *American Journal of Criminal Justice* **40**, 441-55.	Manual collection	Criminology	6 journals	2008-2010	314 articles	34%	Not discussed
Eigenberg & Whalley (2016) *Women and Criminal Justice*, **25**, 130-44.	Manual collection	Criminology	8 journals	2007, 2010 & 2013	998 articles, 2,021 authors	38%	Static
Zettler et al. (2016) *Criminal Justice Studies*, **30**, 30-44.	Manual collection	Criminology	15 journals	1974–2014	11,348 articles	26-33%	Increasing

Yuan et al. (2010) *Journal of Dental Education*, **74**, 372-380.	Manual collection	Dentistry	9 journals	1986, 1990, 1995, 2000, 2005, & 2008	3,556 first and 2,217 last authors	5-42%	Increase
Feramisco et al. (2009). *Journal of the American Academy of Dermatology*, **60**, 63-9.	Manual collection	Dermatology	6 journals	1976, 1986, 1996, 2006.	3070 articles, 11627 authors	31-48%	Increase
Crase (1992) *Death Studies* **16**, 199-209.	Manual collection	Death studies	1 journal	1977-1990	670 authors	39.5%	Not discussed
Lerback and Hanson (2017) *Nature* **541**, 455-7.	Database analysis	Earth and Space Science	American Geophysical Union database (20 journals)	2012-2015	12,564 authors	24%	Not discussed
Martin (2012) *Frontiers in Ecology and the Environment*, **10**: 177-8.	Manual collection	Ecology	1 journal	2011	258 articles, 922 authors	28%	n/a
Fox et al. (2016) *Functional Ecology*, **30**, 126-39.	Manual collection	Ecology	1 journal	2010-2014	551 articles, 14,280 authors	34%	Static
Lockheed & Stein (1980) *Educational Researcher* **9**, 11-5.	Manual collection	Education	6 journals	1973-1978	2,239 articles	4-27%	Static
Zawacki-Richter & von Prümmer (2010) *Journal of Open Learning*, **25**, 95-114.	Manual collection	Education	5 journals	2000-2008	1,059 authors, 695 articles	46%	Not discussed
Robinson et al. (1998) *Contemporary Educational Psychology*, **23**, 331-43.	Manual collection	Educational Psychology	6 journals	Even years 1976-1996	2,270 articles, 4,578 authors	21-66%	Increase/ static
Li et al. (2007) *Academic Emergency Medicine*, **14**, 1194-6.	Manual collection	Emergency Medicine	4 journals	1985, 1995, 1999, 2005	2016 articles, 2,044 first and 2,124 last authors	22-24%	Increase
Ghiasi et al. (2013) *PLoS ONE* **10**, e0145931.	Database analysis	Engineering	All engineering articles on Web of Science	2008-2013	679,338 papers and 974,837 authors	20%	Not discussed
Schrager et al. (2011). *Family Medicine-Kansas City*, **43**, 155.	Manual collection	Family medicine	5 journals	2006-2008	2,126 articles, 2,126 first authors	34%	Static
Long et al. (2015) *Gastrointestinal Endoscopy* **81**, 1440-7.	Manual collection	Gastroenterology	5 journals	1992, 1997, 2002, 2007, 2012	2730 articles, 1,911 first and 1,881 senior authors	14-29%	Increase/ static
Rigg et al. (2012) *The Professional Geographer*, **64**, 491-502.	Manual collection	Geography	6 journals	1995-2006	2,311 articles	22%	Increase

Cunningham & Dillon (1997) *Scientometrics* **39**, 19-27.	Manual collection	Information systems	5 journals	1989-1995	861 articles; 1,021 authors	22%	Not discussed
Porter et al. (2003) *Mental Retardation*, **41**, 1-6.	Manual collection	Intellectual and developmental disability	8 journals	1991-2000	2,972 articles, 7,815 authors	24-62%	Increase/ static
Mihaljević-Brandt et al. (2016) *PLoS ONE* **11**, e0165367.	Database analysis	Mathematics	zbMATH database (>1700 journals)	1970-2016	2.2m articles, 144,253 authors	*c.* 8%	Increase
Burns (2015) *Journal of Information Science Theory and Practice* **3**, 16-30.	Manual collection	Medical and biological sciences	1 journal	2013	49 articles	27%	n/a
Baethge (2008) *Deutsches Arzteblatt International*, **105**, 507-9.	Manual collection	Medicine	1 journal	1957-2008	Not stated	17%	Increase
Filardo et al. (2016) *British Medical Journal* **352**, i847.	Manual collection	Medicine	6 journals	1994-2014	3758 first authors from 3,758 articles	37%	Variable
Jagsi et al. (2006) *New England Journal of Medicine*, **355**, 281-7.	Manual collection	Medicine	6 journals	1970, 1980, 1990, 2000, 2004	3,872 first and 3,377 senior authors	20-30%	Increase
Sidhu et al. (2009) *Journal of the Royal Society of Medicine*, **102**, 337-42.	Manual collection	Medicine	6 journals	1970, 1980, 1990, 2000, 2004	6457 articles, 3,084 first and 3,105 senior authors	17-37%	Variable
Naldi and Parenti (2002) *Technical report EUR 20309, Consiglio Nazionale delle Ricerche.*	Manual collection	Multiple disciplines	157 journals	1995	9,688 papers, 36,239 authors	20%	n/a
Larivière et al. (2013) *Nature* **504**, 211-3.	Database analysis	Multiple disciplines	Web of Science (unstated number of journals)	2008-2012	4.4m papers and 17.8m authors	”Fewer than 30%”	Not discussed
Mauleon et al. (2013) *Scientometrics* **95**, 87-114.	Manual collection	Multiple disciplines	36 journals	1998, 2008	2,945 articles, 9,225 authors	14-44%	Increase
West et al. (2013) *PLoS ONE* **8**, e66212.	Database analysis	Multiple disciplines	JSTOR database (many journals)	1990-2011	2.05 million authors	About 29% (across JSTOR)	Increase
Macaluso et al. (2016) *Academic Medicine*, **91**: 1136-42.	Database analysis	Multiple disciplines	8 *PLoS* journals	2008-2013	85,000 articles	*c.* 45-50%	Not discussed
Berg (2017) *Science* **355**, 329.	Manual collection	Multiple disciplines	1 journal	2015	1950 authors	27%	n/a

Elsevier (2017) *Gender in the Global Research Landscape*, elsevier.com/research-intelligence/ resource-library/gender-report	Database analysis	Multiple disciplines	Scopus database	1996-2000, 2011-2015	Not stated (millions?)	9-65%	Increase
Conley & Stadmark (2012) *Nature* **488**, 590.	Manual collection	*Nature* and *Science* news sections	2 journals	2010 and 2011	Not stated	4-8% in Nature	Not discussed
Editorial (2017) *Nature* **541**, 435.	Manual collection	*Nature* News and Views section	1 journal	2015-2016	Not stated	25%	Increase
Jarema et al. (1999) *Journal of Organizational Behavior Management*, **19**, 85-94.	Manual collection	Organizational Behavior Management	1 journal	1977-1997	632 authors	33%	Increase
McGee et al. (2004) *Journal of Organizational Behavior Management*, **23**, 3-13.	Manual collection	Organizational Behavior Management	4 journals	1978-2000	<2000 authors	25-35%	Increase
Bhattacharyya & Shapiro (2000) *Laryngoscope*, **110**, 358-61.	Manual collection	Otolaryngology	4 journals	1978, 1988, 1998	2,463 articles, 2,133 first authors	11-12%	Increase
Bergeron et al. (2012) *Otolaryngology Head and Neck Surgery*, **147**, 40-3.	Manual collection	Otolaryngology	4 journals	1978, 1988, 1998, 2008	2671 articles, 2,671 first authors	23%	Increase
Brown & Goh (2016) *Social Psychological and Personality Science*, **7**, 437–43.	Manual collection	Personality & social psychology	2 journals	2004-2013	1,094 articles	37-60%	Increase
Dotson (2011) *American Journal of Health-System Pharmacy*, **68**, 1736-9.	Manual collection	Pharmacy	3 journals	1989, 1999, 2009	608 articles	53%	Increase
Kongkiatkamon et al. (2010) *Journal of Prosthodontics*, **19**, 565-70.	Manual collection	Prosthodontics	3 journals	1995, 2000, 2005, 2008	1,202 articles, 451 first and 323 last authors	6-15%	Static
Alonso-Arroyo et al. (2008) *Actas Espanolas de Psiquiatria*, **36**, 314-22.	Manual collection	Psychiatry	1 journal	1999-2006	458 articles, 977 authors	39%	Increase
Amering et al. (2011) *Academic Medicine*, **86**, 946-52.	Manual collection	Psychiatry	3 journals	1994, 2007	1732 articles, 1,732 first authors, 5,526 co-authors	34%	Increase
Erden Aki et al. (2015) *Archives of Neuropsychiatry* **52**, 95-8.	Manual collection	Psychiatry	Turkish first authors	1980-2009	1,961 articles	35-37%	Static
White (1985) *American Psychologist*, **40**, 527-30.	Manual collection	Psychology	14 journals	1972, 1982	First authors, not stated	12-46%	Increase
McSweeney et al. (2002) *The Behavior Analyst* **25**, 37-44.	Manual collection	Psychology	17 journals	1978-1997	Not stated	17-65%	Increase

Slack et al. (1996) *Public Administration Review*, **56**, 453-8.	Manual collection	Public Administration	1 journal	1940-1995	3,207 authors	9.7%	Increase
White (1997) *Research in Science Education* **27**, 215–21.	Manual collection	Science Education	1 journal	1975, 1985 & 1995	129 authors	*c.* 30%	Increase
Kurichi et al. (2005) *Archives of Surgery*, **140**, 1074-7.	Manual collection	Surgery	2 journals	Odd years 1985-2003	830 articles	20-27%	Increase
Nunkoo et al. (2017) *Annals of Tourism Research* **63**, 207-10.	Manual collection	Tourism Research	1 journal	1990-2015	2,272 authors	*c.* 40%	Increase
