# Supplementary material for: The gender gap in science: How long until women are equally represented?
Source: PLoS Biol. 2018 Apr 19;16(4):e2004956. doi: 10.1371/journal.pbio.2004956 (PMC5908072; doi:10.1371/journal.pbio.2004956)
Supplement: S3 Table — Journal and discipline each explained about a quarter of the variation, while country explained 11%. The random slopes and intercepts were weakly negatively correlated for journal and discipline, reflecting the fact that percentage of women authors is increasing in most fields with a male-biased gender ratio and is decreasing (or increasingly more slowly) in most fields with a female-biased gender ratio. (PDF) [file pbio.2004956.s024.pdf]

Random effect	Component	Variance explained	% variance
Journal	Intercept	104.38	24.5
	Slope	2.14	0.5
	Slope-intercept correlation	-0.20	
Discipline	Intercept	114.11	26.8
	Slope	0.44	0.1
	Slope-intercept correlation	-0.11	
Country	Intercept	46.33	10.9
	Slope	1.35	0.3
	Slope-intercept correlation	0.02	
Residual	-	156.62	36.8
