# Supplementary material for: The gender gap in science: How long until women are equally represented?
Source: PLoS Biol. 2018 Apr 19;16(4):e2004956. doi: 10.1371/journal.pbio.2004956 (PMC5908072; doi:10.1371/journal.pbio.2004956)
Supplement: S4 Table — The ‘Use last for single’ simulation generated the expected author GR of invited authors under the null hypothesis (i.e., that there is no difference in GR between invited and noninvited authors) using our estimates of how journal, authorship position, and publication date affect GR. For cases where we had no estimate of the GR of single authors, we assumed it was the same as the GR of last authors, increasing the sample size of the test. The ‘Complete records only’ method is the same, but instead of filling in missing data, it simply excludes cases for which we did not have an estimate of the GR for all authorship positions. The ‘Use last for all’ method assumes that the GR of invited authors is expected to be the same as the GR for last authors (even among first and middle authors on invited papers)—this captures our expectation that invited authors will tend to be drawn from the pool of senior researchers (which is male-biased), rather than the total population of researchers. The final method is the same, except that it assumes that the GR should be equivalent to that estimated for single authors. In all simulations, the null expected gender ratio for all invited papers was randomly generated 10,000 times, then compared to the observed gender ratio as both an absolute difference and a fold difference (p < 0.00001 for all four models). The 95% quantiles of this differences were used to estimate the 95% confidence limits on the difference. The final columns give the sample sizes for each method in terms of authors, papers, and journals. GR, gender ratio. (PDF) [file pbio.2004956.s025.pdf]

Simulation method	% women invited authors (Expected)	Absolute difference (95% CIs)	Fold difference (95% CIs)	Authors	Papers	Journals
Use last for single	15.9 (30.0)	14.2% (12.5-15.8)	1.9 (1.8-2.0)	2354	3063	89
Complete records only	14.1 (24.0)	9.8% (8.2-11.4)	1.7 (1.6-1.8)	1781	2490	53
Use last for all	15.8 (29.2)	13.4% (11.8-15.1)	1.8 (1.7-2)	2357	3067	90
Use single for all	8.4 (17.8)	9.4% (7.6-11.1)	2.1 (1.9-2.3)	1505	1823	6
