# Supplementary material for: The gender gap in science: How long until women are equally represented?
Source: PLoS Biol. 2018 Apr 19;16(4):e2004956. doi: 10.1371/journal.pbio.2004956 (PMC5908072; doi:10.1371/journal.pbio.2004956)
Supplement: S5 Table — We identified the top model by comparing the AICc scores of the full model (containing the 3 predictors and all 2- and 3-way interaction terms) and all possible simpler models. ‘Relative impact factor’ is a continuous predictor, defined as the residuals from a model with Log10 IF as the response variable and research discipline as a random effect (i.e., it gives the IF after adjusting for the differences in impact factor that exist between disciplines). ‘Review journal’ and ‘OA journal’ are both 2-level factors describing whether the focal variable is a review journal or an Open Access journal. AICc, corrected Akaike Information Criterion; IF, impact factor. (PDF) [file pbio.2004956.s026.pdf]

	Sum Sq	df	F	p
Intercept	0.06	1	0.00	0.98
Relative impact factor	2289	1	33.4	<0.0001
Review journal	368	1	5.36	0.021
OA journal	100	1	1.47	0.226
Review $\times$ OA	654	1	9.54	0.002
Residuals	186199	2716		
