# Supplementary material for: The gender gap in science: How long until women are equally represented?
Source: PLoS Biol. 2018 Apr 19;16(4):e2004956. doi: 10.1371/journal.pbio.2004956 (PMC5908072; doi:10.1371/journal.pbio.2004956)
Supplement: S2 Table — Predictors that significantly correlated with percentage of women authors are shown in bold. ‘Authorship position’ is a 4-level factor with the levels first, last, middle, or single author. The remaining predictors are continuous variables, though publication date was rounded off to the nearest year. (PDF) [file pbio.2004956.s023.pdf]

	χ^2	df	p
Intercept	991.82	1	<0.0001
**Publication date**	104.41	1	<0.0001
**Authorship position**	78629.52	3	<0.0001
Mean life expectancy	0.18	1	0.67
**Mean years of school (both genders)**	8.03	1	0.0046
**Per capita gross national income**	19.64	1	<0.0001
Adolescent birth rate	2.34	1	0.13
% women in parliament	0.00	1	0.98
Gender gap in education	0.51	1	0.47
Gender gap in the labour force	0.51	1	0.47
